# Supplementary material for: Calcium scoring during 18F-FDG PET/CT in cancer indications: Improving cardiovascular risk stratification and prevention
Source: PLoS One. 2025 Oct 30;20(10):e0335249. doi: 10.1371/journal.pone.0335249 (PMC12574858; doi:10.1371/journal.pone.0335249)
Supplement: S1 Table — CAC, coronary artery calcium; CHD, coronary heart disease; CVD, cardiovascular disease. (DOCX) [file pone.0335249.s001.docx]

**S1 Table. Comparison of median CAC scores between patient subgroups using the Wilcoxon rank-sum test.**

| Variables | CAC median (IQR) | P-value |
| --- | --- | --- |
| Male (n=69)  Female (n=120) | 114 (20-421)  10 (0-105) | P < 0.001 |
| Non-diabetics (n=162)  Diabetics (n=27) | 22 (0-138)  158 (12-1783) | P = 0.002 |
| No hypertension (n=109) | 9 (0-95) | P < 0.001 |
| Hypertension (n=80) | 111 (10-365) |  |
| No dyslipidemia (n=133) | 15 (0-136) | P < 0.001 |
| Dyslipidemia (n=56) | 104 (18-391) |  |
| No family history of CHD (n=129) | 28 (1-196) | P = 0.828 |
| Family history of CHD (n=59) | 34 (1-139) |  |
| Never smoke (n=101) | 20 (0-138) | P = 0.078 |
| Former or active smoker (n=88) | 42 (5-292) |  |
| History of CVD (n=13) | 3031 (1906-3788) | P < 0.001 |
| No history of CVD (n=176) | 22 (0-126) |  |

CAC, coronary artery calcium; CHD, coronary heart disease; CVD, cardiovascular disease
