# Supplementary figures and images for: Calcium scoring during 18F-FDG PET/CT in cancer indications: Improving cardiovascular risk stratification and prevention
Source: PLoS One. 2025 Oct 30;20(10):e0335249. doi: 10.1371/journal.pone.0335249 (PMC12574858; doi:10.1371/journal.pone.0335249)

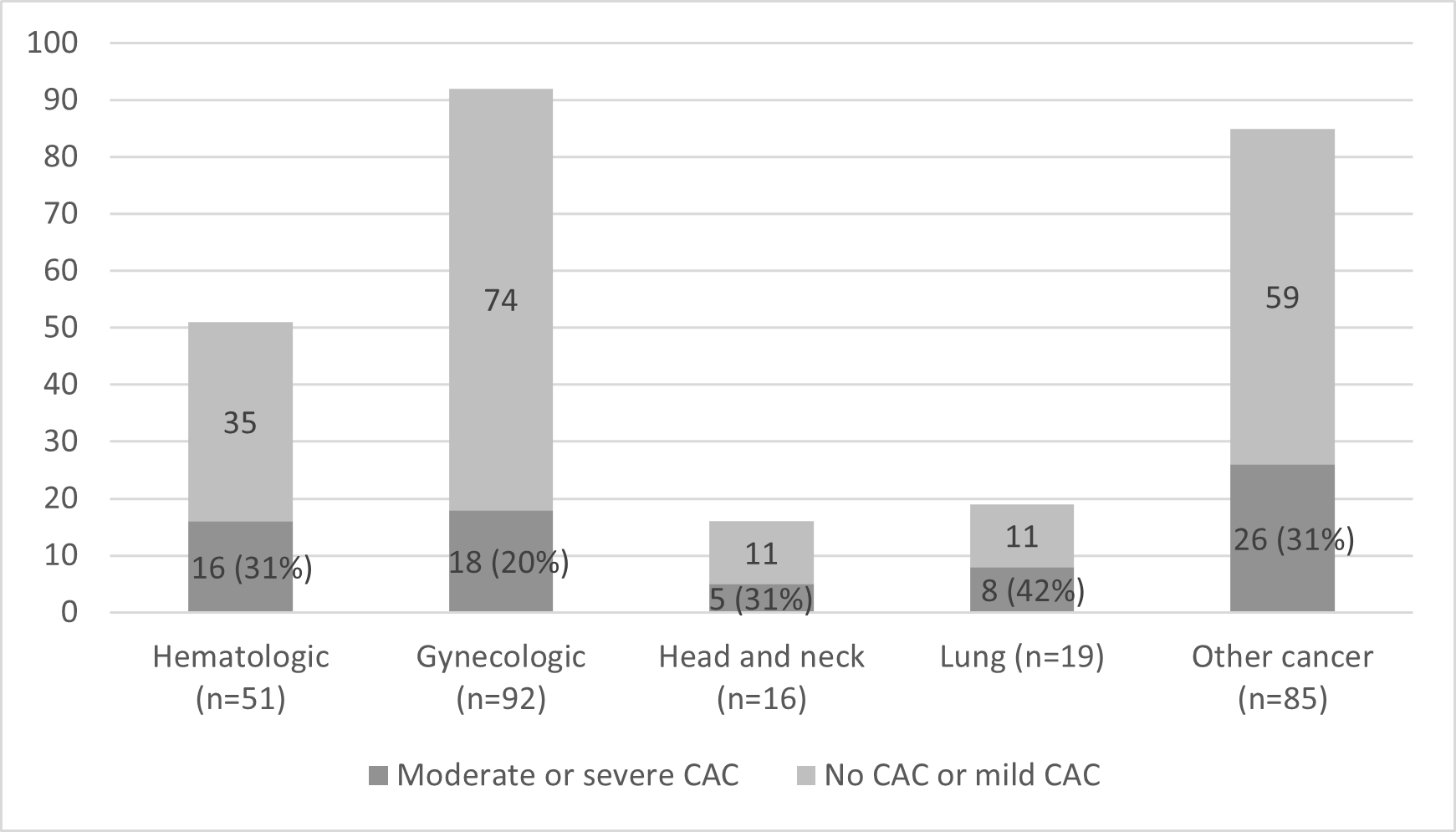

Supplement: S1 Fig — This bar chart illustrates the proportion of patients with moderate to severe calcifications (dark grey), indicating a high cardiovascular risk according to the CAC score, within each patient subgroup defined by cancer type. The proportion is significantly higher in the “lung cancer” subgroup (p < 0.05). CAC, coronary artery calcium. (TIF) [file pone.0335249.s002.tif]
